# 

**Published:** 1893-07-29

**Authors:** 


### CONTENTS.

The "Pall Mall Gazette" and the London Hospital - 273
Around the Hospitals - 274
Medical History from the Earliest Times. By E. T. Withington, M.A., M.B.Oxon. LXIV.—The Origin of Modern Medicine - 275
Contemporary Theory and Research - 276
A Physiological Lantern. I. "Light, More Light" - 277
Gleanings in Science and Medicine - 278
Annotations.—A Doctor, not a Doormat—Thin Skins—A Striking Experiment in Vaccination - 279
The Institutional Workshop—
Hospital Construction.—Royal Victoria Hospital, Montreal - 285
Festival Dinners and Meetings.—King's College Hospital - 287
Construction Notes. - 288
Notes and News - 280
The Hospital Clinic—
Royal Orthopædic Hospital.—Treatment of the Lateral Curvature of the Spine - 281
Paddington Green Children's Hospital.—The Treatment of Pleurisy and Empyema - 282
The Bristol Eye Hospital.—Treatment of Glaucoma - 283
New Appliances and Things Medical.—Waterproof Sheeting—Carbo-Eucalyptine Volatile Sanitary Tablets—"Sanitas"—"Franz Josef" Natural Purgative Water - 284
Editor's Letter-Box - 288
Scraps and Gleanings - 288
The Book World of Medicine and Science - vii
Medical Vacancies, Appointments, &c. - ix

### EXTRA SUPPLEMENT.—THE NURSING MIRROR.

News from the Nursing World.—Our Christmas Competitions—Uniform versus Registration—In Service but not of Service—An Awkward Omission—Are Curls Comforts?—Curiosities in Disease—Poor Economy—An Underpaid Amateur—Festivities at Worksop—Queen's Nurses—Sea-shells—Proposed Nurse Training in Ireland—Short Items - clxx

Sanitation for Nurses. By A Sanitary Inspector XV. Apparatus for Disinfection - clxxiii

Educational Standards for Nurses. By Miss Isabel A. Hampton. III.—Work to be Accomplished in the Future - clxxiv

Our American Letter. By A Trained Nurse - clxxv

Presentations - clxxv

Everybody's Opinion.—Hospitals for Soldiers' Wives—A Holiday in Cornwall—Australia or New Zealand? - clxxvi

Novelties for Nurses.—Canfield Waterproof Specialities - clxxvi

Appointments—Minor Appointments - clxxvi

The Royal British Nurses' Association - clxxvi

Press Notices - clxxvi

Wants and Workers - clxxvi

A Novel Competition for Nurses - clxxvii

For Reading to the Sick.—"Mutual Love" - clxxvii

The Muses' Looking-Glass - clxxviii

Notes and Queries - clxxviii

The Book World for Women and Nurses - clxxix

